# The relationship between self-efficacy and error orientation of nursing students during clinical internships: a cross-sectional study

**DOI:** 10.3389/fpubh.2024.1432962

**Published:** 2024-07-12

**Authors:** Yuanli Guo, Wenfeng Fan, Xiaofang Dong, Caixia Yang, Min Wang, Huanhuan Gao, Peihua Lv, Keke Ma

**Affiliations:** ^1^The Neurology Department, The First Affiliated Hospital of Zhengzhou University, Zhengzhou, Henan, China; ^2^School of Nursing and Health, Zhengzhou University, Zhengzhou, Henan, China; ^3^The Nursing Department, The First Affiliated Hospital of Zhengzhou University, Zhengzhou, Henan, China

**Keywords:** error orientation, internship, motivation, nursing errors, nursing student, security, self-efficacy

## Abstract

**Background:**

Nursing students often make clinical errors due to their limited clinical experience and their orientation toward errors, revealing their attitude and behavioral tendencies regarding nursing errors. Understanding how self-efficacy, motivation, and a sense of security influence the error orientation of nursing students is important for developing strategies to enhance their error orientation.

**Objectives:**

This study aimed to explore the relationship between self-efficacy, motivation, and error orientation of nursing students during clinical internships.

**Method:**

This was a cross-sectional study. An electronic questionnaire was distributed to nursing students from 14 September 2023 to 30 September at a comprehensive tertiary A teaching hospital in Zhengzhou, Henan province. The instruments used in this study included the General Information Questionnaire, General Self-efficacy Scale, Achievement Motives Scale, Security Scale, and Error Orientation Scale. Statistical Product and Service Software Automatically (SPSSAU) was used to perform statistical description, mediation analysis, and moderated mediation analyses.

**Results:**

A total of 510 nursing students were included in this study. The motivation for success and failure-escaping fully mediated the relationships between self-efficacy and error orientation of nursing students, with a mediation effect of 0.101 (95% CI: 0.058–0.144). The security of nursing students moderated both the direct effect of this model and the indirect effect of motivation for failure-escaping. When security was high, the self-efficacy of nursing students was positively correlated with their error orientation, with an effect of 0.059 (95% CI: 0.003~0.116). When security was high, the moderation effect was significant, with an effect of −0.012 (95% CI: −0.026~-0.002). However, at low and median levels of security, the mediation effect was non-existent.

**Conclusion:**

The motivation for success and failure escaping play different roles in the paths between self-efficacy and error orientation. Clinical nursing teachers should take measures to enhance the motivation for success but reduce the failure-escaping motivation to improve the error orientation of nursing students. Additionally, it is crucial to pay attention to and improve the sense of security of students during clinical internships.

## 1 Introduction

A clinical internship refers to a period of training that nursing students must complete before becoming qualified professional nurses (1). However, as nursing students do not provide nursing care to patients before their internships, they find various operations in hospitals unfamiliar and extremely challenging. As a result, some medication errors inevitably occur during their clinical practice, which may lead to adverse events or near misses, jeopardizing patient safety (2). A survey reported that 17.8% of nursing students in China (3) experience adverse events. In an observational retrospective longitudinal study that included 4,284 undergraduate nursing students in Spain, 38.2% of the participants were reported to have caused adverse events during their clinical practice (4). Adverse events not only harm patient safety but also create negative emotional experiences for nursing students, which may have a significant impact on their future work (2). Therefore, it is necessary to conduct in-depth research on how nursing students respond to adverse events, which is called “error orientation.” Understanding the concept of error orientation can guide nursing leaders to change policies, practices, and training programs within healthcare settings to help students learn from mistakes and develop strategies to minimize errors in their practice, ultimately leading to continuous professional development and improvement in the quality of care provided by nursing students. Gradisnik et al. (5) explored how nursing students handle patient safety incidents during their clinical practice. The findings included an examination of their emotional responses, the actions taken, the factors contributing to these incidents, and the resulting consequences. However, there have been few quantitative research reports on the attitudes and learning behaviors of nursing students when encountering such incidents related to medical errors (adverse events or patient safety incidents).

“Error” can be defined as an unintentional behavior of an individual that deviates from expected results due to a lack of certain knowledge, skills, or an inability to provide timely feedback (6). Frese and Keith (7) observed that individuals will take some actions to respond to their errors with a certain inclination, which is known as “error orientation.” Wei and Hisrich (8) defined error orientation as a cognitive strategy adopted by individuals for error management from psychological and behavioral perspectives. An individual with a positive error orientation is more likely to seek help from colleagues when an error occurs. This practice promotes internal learning and knowledge transfer within the organization and facilitates reporting more errors, driving the organization to improve its efficiency in handling errors (9). Thus, a nursing student with a positive error orientation may be more proactive in reporting errors and learning from them to promote personal progress. Furthermore, it enables clinical instructors or nursing managers to address errors promptly, thereby preventing their escalation and allowing for the development of targeted training aimed at reducing the recurrence of similar errors. Therefore, it is critical to understand the level of nursing students' error orientation and the factors influencing it.

**Figure 1:**
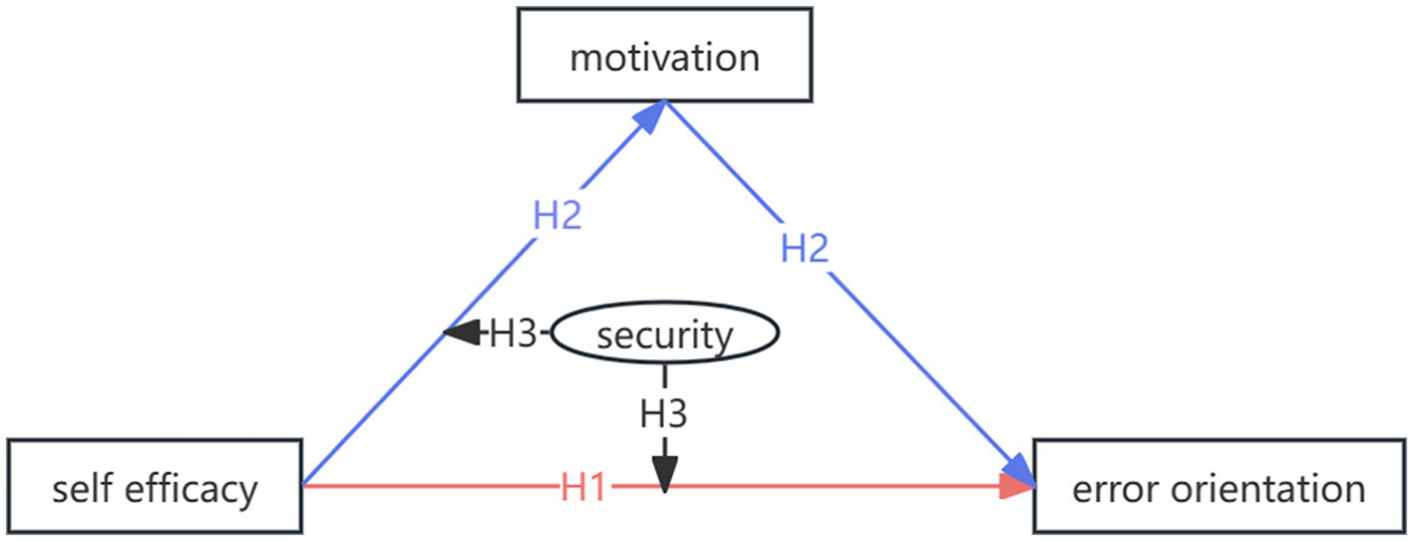
The theoretical framework and hypothesis of this study.

Self-efficacy is an individual's belief in their ability to successfully perform specific behaviors (10), which is identified to correlate with error orientation; that is, individuals with higher self-efficacy tend to handle errors more rationally and learn more from them (11). Therefore, we propose the first hypothesis that the self-efficacy of nursing students is correlated with their error orientation. According to self-efficacy theory, the motivation process follows one of the four paths (process of cognition, motivation, emotion, and selection), while self-efficacy influences the attitude and behavior of an individual (12). Therefore, for nursing students engaged in clinical internships, their motivation might act as a mediating variable between their self-efficacy and their attitude and behavior toward errors, which is the second hypothesis formulated in this study. Security in the learning environment for nursing students means that they do not have to worry about negative consequences or the risk of embarrassment, condemnation, and blame, allowing them to learn with peace of mind and composure (13). The environment of a clinical internship has more risks compared to school learning because students have to face real nursing practice instead of simulations, in which slight carelessness can result in nursing errors. A prior study demonstrated that the self-efficacy of nursing students was positively related to their sense of security (14). Accordingly, our third hypothesis posits that different levels of security among students will affect the mediation of motivation between self-efficacy and error orientation. Thus, to identify conditions or factors that strengthen or weaken the link between these variables and to provide a more nuanced understanding of how nursing self-efficacy, motivation, and sense of safety influence error orientations, we decided to adopt a moderated mediation analysis. The theoretical framework is shown in Figure 1.

## 2 Objectives

This study aimed to explore the mediation effect of motivation and the moderation effect of security between self-efficacy and error orientation of nursing students engaged in clinical internships. For this purpose, we proposed the following hypotheses:

**H1**: The self-efficacy of nursing students is correlated with their error orientation.

**H2:** Motivation is a mediating variable in the association between the self-efficacy and the error orientation of nursing students.

**H3**: The security of nursing students moderates the mediation effect of motivation between self-efficacy and error orientation in H1.

## 3 Design, sample, and settings

This is a cross-sectional study conducted from 14 September 2023 to 30 September at a comprehensive tertiary A teaching hospital in Zhengzhou, Henan province, which has 279 wards in 120 clinical departments in 4 districts located in the east, south, and north of Zhengzhou. The hospital recruits approximately 600 nursing students from all over the country for clinical internships every year. The study's inclusion criteria were as follows: participants should be at least 18 years old and currently enrolled in a clinical internship at this sampling hospital's clinical department. Students who participated in programs related to nursing errors were excluded. Convenient sampling was used in this study because the hospital where the research was conducted is the largest teaching hospital in the province, so the interns recruited come from various regions of the province, making the sample more representative. The survey was distributed in the form of an electronic questionnaire on the “Questionnaire Star (wjx.cn)” platform. Researchers created and distributed questionnaires, exporting data to personal accounts. The survey content did not include the names of the participants, guaranteeing the anonymity of research subjects and the security of the data, as access was restricted to only authorized individuals. After negotiation with the nursing department, the questionnaire link was sent to the head nurses and clinical head teachers of each department, who then sent it to the nursing interns working in their department via WeChat. After the interns completed the questionnaire, they took a screenshot of the “survey completed” page and sent it back to ensure a response rate. According to the Kendell sample size estimation method (15), the optimal sample size should be 15 times the number of variables. There were 14 variables in this study and, therefore, the smallest sample size required was 263, considering 20% of the inefficient samples.

## 4 Tools

The tools used in this study included the General Information Questionnaire,

Achievement Motives Scale (AMS), Security Scale (SS), and Error Orientation Scale (EOS). The author contacted the original author before using the AMS, SS, and EOS and informed them that these tools would be used in the form of an electronic questionnaire, obtaining the original author's consent.

### 4.1 General information questionnaire

The researchers designed the General Information Questionnaire according to the study's aim. The questions related to the respondent's age, sex, school level, personal experience with nursing errors, whether they had witnessed nursing errors, and the duration of their clinical internship.

### 4.2 General self-efficacy scale (GSES)

The GSES, Sinicized by Hu (16) in 2014, was used to assess nursing students' self-efficacy. This scale comprises 10 items, and a 4-point Likert scale, ranging from 1 (totally wrong) to 4 (totally right), was adopted. A higher total score indicates higher self-efficacy. The value of Cronbach's α was 0.930 in this study.

### 4.3 Achievement motives scale (AMS)

In 1970, Gjesme and Nygard compiled the Achievement Motives Scale, which was later adapted into a Chinese version by Ye and Hagtvet in 1992 (17). This scale comprises 30 items categorized into two dimensions: motivation for success (FS) and failure-escaping (FE). Each item is rated on a 4-point Likert scale, ranging from 1 (completely inconsistent) to 4 (completely consistent). The score of each dimension was the total score of all the items in this dimension. A higher score indicates a stronger level of achievement motivation. In this study, the scores of two dimensions were calculated and analyzed as two independent variables, and the value of Cronbach's α was 0.935.

### 4.4 Security scale (SS)

The Security Scale was developed by Zhong et al. (18) in 2004, and it includes 16 items in two dimensions. Each item is scored on a 5-point Likert scale according to the response from 1 (strongly disagree) to 5 (strongly agree). The total score was estimated as the sum of each item's score, and a higher total score means lower security. Cronbach's α value was estimated to be 0.906.

### 4.5 Error orientation scale (EOS)

The error orientation scale was developed by Rybowiak et al. (19) in 1999, which includes 37 items in 8 dimensions (error competence, learning from errors, error risk-taking, error strain, error anticipation, covering up errors, error communication, and thinking about errors). The 5-point Likert scale was adopted, ranging from 1 (strongly disagree) to 5 (strongly agree). The items in error strain and covering up error dimensions were scored in reverse. A higher total score indicated better error orientation. Cronbach's α was estimated to be 0.883.

## 5 Data collection and ethical considerations

An electronic questionnaire was distributed online, and the link was sent to the WeChat groups of nursing students and the clinical head teachers. The clinical head teacher of each department supervised interns in completing the questionnaire. The questionnaires with a filling time of less than 300 s were regarded as invalid and were deleted according to the online questionnaire collection system backend, and other collected questionnaires were checked by researchers. The questionnaires with obvious logic-related errors and those with more than 90% of the same responses were also regarded as invalid. The design and implementation of this study complied with the requirements of the Declaration of Helsinki (20). The informed consent to participate in this survey from the nursing students was obtained before the questionnaire was filled out. This survey was approved by the Ethics Committee of the First Affiliated Hospital of Zhengzhou University.

## 6 Data analysis

An online data analysis system, SPSSAU (https://spssau.com/indexs.html), was used in this study. For quantitative data, normality testing was performed using the Shapiro–Wilk test of normality and normal Q–Q plots. The data that conformed to a normal distribution were represented by means and standard deviations. The median and percentile were used for the data that did not conform to a normal distribution. For counting data, frequency and percentage were used for representation. The association between variables was analyzed using the Pearson correlation analysis. The moderated mediation effect was analyzed using the PROCESS macro in SPSSAU, and model 59 was adopted. The bootstrap sampling frequency was set at 1,000, and the low/high level of the moderator variable was Mean±1SD. The exploratory factor analysis (EFA) approach was adopted to test the common method bias, and the results with more than one factor having eigenvalues greater than one and the variance explanatory power of the maximum factor <40% were regarded as a non-serious common method bias (21). Before conducting mediation and moderation analyses, all variables were centralized to avoid interference from multicollinearity.

## 7 Results

### 7.1 The general information of participants

Out of the 564 students enrolled this year, 518 (91.8%) nursing students completed the electronic questionnaire, and 510 valid questionnaires were included for data analysis. Among the respondents, 62 (12.16%) were men, and 448 (87.84%) were women, resulting in a questionnaire efficiency rate of 98.5%. A total of 72 students (14.12%) reported having made nursing errors themselves, and 286 students (56.08%) had witnessed nursing errors made by others. The detailed general information is presented in Table 1.

**Table 1:** The general information of participants (*N* = 510).

**Variables**	***N* (%)/Mean (SD)**
**Sex**	
Male	62 (12.16)
Female	448 (87.84)
**School degree**	
Project 985/211 universities	22 (4.31)
Universities with “Double First-Class” disciplines	2 (0.39)
The first batch of universities	42 (8.24)
The second batch of universities	140 (27.45)
Academic college	298 (58.43)
Other college	4 (0.78)
**Had made a nursing error**	
Yes	72 (14.12)
No	438 (85.88)
**Had seen a nursing error**	
Yes	286 (56.08)
No	224 (43.92)
Knowledge about nursing errors	22.85 (5.46)
Age	20.84 (1.23)
Weeks of clinical internship	12 (3, 20)
Weeks of training related to nursing errors	3 (1, 5)

### 7.2 Results of the common method bias test

The results of EFA for all items revealed that there were 14 factors with eigenvalues >1, and the maximum variance explanatory rate was 23.679% (<40%), which meant there was no serious common method bias.

### 7.3 Scores of self-efficacy, motivation, security, and error orientation of nursing students

The results showed that the scores of self-efficacy, pursuit of success motivation, avoidance of failure motivation, security, and error orientation of nursing students were 28.44 ± 4.80, 43.45 ± 7.65, 40.24 ± 7.99, 42.34 ± 7.65, and 131.59 ± 30.68, respectively. Among the seven dimensions of error orientation, the “thinking about errors” dimension had the highest average score of 4.26 ± 0.64, and the “covering up errors” dimension had the lowest average score of 2.15 ± 1.06.

### 7.4 Pearson's correlation analysis of self-efficacy, motivation, security, and error orientation of nursing students

The results showed that the security (r = −0.219, *p* < 0.01) and the failure-escaping motivation (r = −0.145, *p* < 0.01) of nursing students exhibited a negative correlation with their error orientation, and the self-efficacy (r = 0.316, *p* < 0.01) and the motivation of pursuit of success (r = 0.363, *p* < 0.01) demonstrated a positive correlation with their error orientation (Table 2).

**Table 2:** Results of Pearson's correlation analysis (*n* = 510).

	**GESE**	**SS**	**FS**	**FE**	**EOS**
GESE	1				
SS	0.144^**^	1			
FS	0.671^**^	0.150^**^	1		
FE	0.180^**^	0.583^**^	/	1	
EOS	0.316^**^	−0.219^**^	0.363^**^	−0.145^**^	1

^**^p <0.01.

### 7.5 The analysis of the mediation effect of motivation and the moderation effect of security

#### 7.5.1 The mediation effect of motivation

The mediation effect was first analyzed in accordance with the method proposed by Wen et al. (21). After controlling the variables in general information, the results showed that failure-escaping motivation and pursuit of success had a complete mediation effect on the relationship between self-efficacy and error orientation of nursing students, and the total effect was 0.101 (95% CI: 0.058–0.144). The self-efficacy of nursing students was positively correlated with their motivation for success (β = 0.637) and failure-escaping (β = 0.191).

#### 7.5.2 The moderated mediation effect analysis

**Figure 2:**
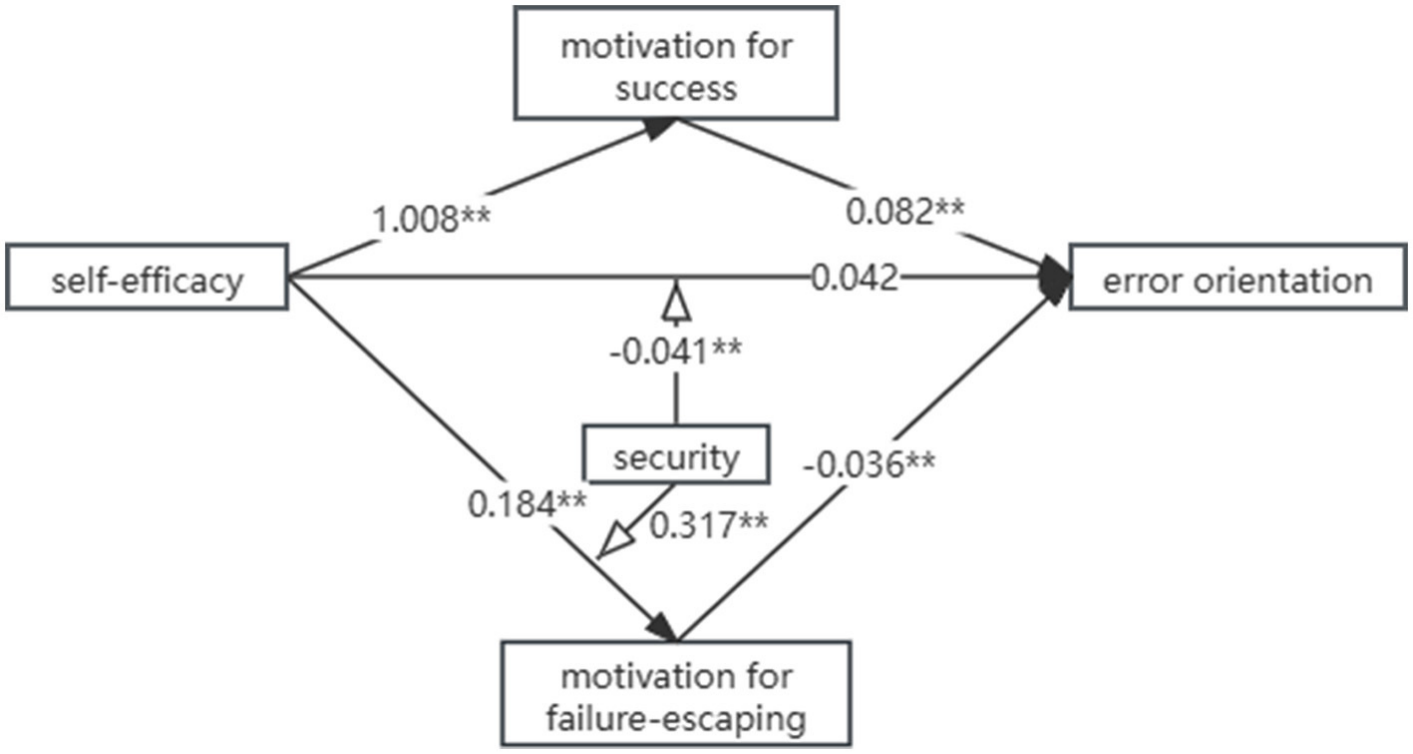
The pathway and coefficient among self-efficacy, motivation, security, and error orientation. **p <0.01.

Model 8 was used to analyze the moderation effect of security in the mediation model among the self-efficacy, motivation, and error orientation of nursing students. The results revealed that the direct effect was significantly different at low, median, and high levels of security. More particularly, when security was at low and median levels, self-efficacy had no significant correlation with the error orientation of nursing students; however, when security was at a high level, the self-efficacy of nursing students was positively correlated with their error orientation (Table 3). In the analysis of the moderated effect on the indirect effect of the mediation model (Table 4), the results showed that security moderated the mediation effect of failure-escaping motivation between the self-efficacy and the error orientation of nursing students. Specifically, when security was at a high level, the 95% CI of the mediation effect of failure-escaping motivation between self-efficacy and error orientation was −0.026 to −0.002, which meant the moderation effect was significant; however, when at low and median levels of security, the 95% CI of the mediation effect of failure-escaping motivation was ~0.012 to ~0.010 and ~0.018 to ~0.001, respectively, which meant that the mediation effect was non-existent. As for the motivation for success, the results showed that the mediation effect was not significant at low, medium, or high levels of security. The remaining results are detailed in Table 5, and the pathway is shown in Figure 2.

**Table 3:** Results of the conditional direct effect.

**Level**	**SS (centralization)**	**Effect**	**SE**	**t**	***p***	**LLCI**	**ULCI**
X−1SD	−13.654	0.024	0.030	0.816	0.415	−0.034	0.083
Mean	0.000	0.042	0.026	1.600	0.110	−0.009	0.093
X+1SD	13.654	0.059	0.029	2.050	0.041	0.003	0.116

LLCI refers to the lower limit of the 95% confidence interval. ULCI refers to the upper lower limit of the 95% confidence interval.

**Table 4:** Results of conditional indirect effect.

**Mediation variables**	**Level**	**SS (centralization)**	**Effect**	**BootSE**	**BootLLCI**	**BootULCI**
FS	X−1SD	−13.654	0.083	0.019	0.046	0.121
	Mean	0.000	0.082	0.019	0.045	0.120
	X+1SD	13.654	0.082	0.019	0.045	0.122
FE	X−1SD	−13.654	−0.001	0.005	−0.012	0.010
	Mean	0.000	−0.007	0.005	−0.018	0.001
	X+1SD	13.654	−0.012	0.006	−0.026	−0.002

LLCI refers to the lower limit of the 95% confidence interval. ULCI refers to the upper lower limit of the 95% confidence interval.

**Table 5:** The regression model.

	**EOS**				**FS**				**FE**			
	β	**SE**	**t**	***p***	β	**SE**	**t**	***p***	β	**SE**	**t**	***p***
Constant	−2.653	2.187	−1.213	0.226	12.853	5.931	2.167	0.031^*^	−20.362	6.785	−3.001	0.003^**^
GESE	0.042	0.026	1.600	0.110	1.008	0.056	17.837	0.000^**^	0.184	0.065	2.842	0.005^**^
SS	−0.041	0.009	−4.835	0.000^**^	0.027	0.020	1.336	0.182	0.317	0.023	13.980	0.000^**^
GESE^*^SS	0.001	0.001	1.297	0.195	−0.000	0.003	−0.117	0.907	0.011	0.003	3.556	0.000^**^
Knowledge about NE	0.129	0.018	7.216	0.000^**^	0.166	0.049	3.419	0.001^**^	−0.004	0.056	−0.071	0.944
Sex	−0.033	0.289	−0.112	0.910	−0.938	0.778	−1.204	0.229	3.984	0.891	4.473	0.000^**^
Age	0.055	0.085	0.653	0.514	−0.780	0.229	−3.406	0.001^**^	0.661	0.262	2.523	0.012^*^
School level	−0.056	0.068	−0.819	0.413	0.247	0.187	1.320	0.188	−0.116	0.214	−0.541	0.589
Training duration (week)	0.000	0.001	0.597	0.551	0.003	0.001	1.834	0.067	−0.004	0.002	−2.145	0.032^*^
Clinical practice duration (week)	−0.003	0.011	−0.306	0.759	−0.014	0.029	−0.484	0.628	−0.005	0.034	−0.148	0.882
Had happened NE	−0.223	0.332	−0.672	0.502	0.872	0.915	0.953	0.341	−0.232	1.047	−0.222	0.824
Had seen or heard NE	−0.409	0.205	−1.993	0.047^*^	−0.927	0.564	−0.642	0.101	0.250	0.646	0.387	0.699
FS	0.082	0.017	4.919	0.000^**^								
FE	−0.036	0.015	−2.495	0.013^*^								
*R* ^2^	0.316				0.501				0.401			
Adjusted *R* ^2^	0.296				0.489				0.387			
*F*	*F* = 17.589, *p* < 0.001				*F* = 45.471, *p* < 0.001				*F* = 30.345, *p* < 0.001			

^*^p <0.05; ^**^p <0.01.

## 8 Discussion

Error orientation refers to the attitude, cognition, and behavior toward errors. A good error orientation can enable nursing students to continuously grow through various errors in clinical practice, which is crucial for interns who are about to become professional nursing staff. While previous studies have investigated the error orientation of nursing students, this study is the first to focus on the error orientation of nursing students in clinical practice and analyze its relationship with self-efficacy, security, and motivation. These findings can provide good guidance for the development of intervention measures to improve the error orientation of nursing students in the future.

The results showed that the motivation for success and failure escaping played different roles in the relationship between the self-efficacy and the error orientation of nursing students. Specifically, the motivation for success mediated the positive effect between self-efficacy and error orientation, while failure-escaping motivation masked this positive effect. Previous studies have demonstrated a positive correlation between self-efficacy and achievement motivation in student (22), but they did not separately analyze the motivation for success and failure escaping. Bandura et al. (23) demonstrated that individuals with stronger self-efficacy are more eager to succeed and more likely to engage in challenging tasks, even if there is a possibility of failure, which meant that the motivation for success and failure escaping may be two variables that counterbalance each other. However, in this study, we found that the nursing students with stronger self-efficacy had not only higher motivation for success but also higher failure-escaping motivation, which appeared to contradict a previous study. We attribute this difference to the specific context of the research. In a clinical internship, achievement means not only finishing challenging tasks but also ensuring no harm to patients, which might increase their failure-escaping motivation and require them to keep a distance from challenges. The findings from Fan's study (24) support this explanation, which found that nursing students with high success-high failure-escaping motivation had stronger self-efficacy compared to those in the middle success–middle failure-escaping group and the low success–middle failure-escaping group during their internship. Moreover, the bigger path coefficient of motivation for success (1.008^**^) compared to failure-escaping (0.184^**^) indicates the greater positive influence of self-efficacy on the former, which inspired the nursing educator to believe that improving self-efficacy was advisable to enhance the error orientation of nursing students in clinical practice.

The contrasting effects of motivation for success and failure-escaping between self-efficacy and error orientation found in this study might be due to the behavior of students with higher motivation for success. These students are more likely to address unfamiliar nursing practices, exposing them to more situations of patient safety threats and errors. This exposure contributes to their lessons learned and professional growth (25), equipping them with more knowledge and skills to cope with errors. Conversely, motivation for failure-escaping could deprive students of many opportunities to learn from practice. Coupled with the lack of clinical error education in the classroom (26), this makes it difficult for nursing students to adopt a positive attitude, cognition, and behavior when handling errors, resulting in poor error orientation.

According to the results, self-efficacy had no significant correlation with the error orientation of nursing students during internships, which seemed to contradict our first hypothesis. However, when we introduced security as a moderating variable, self-efficacy was positively correlated with the nursing students' error orientation (β = 0.059, CI: 0.003–0.116) when the security score was high (high score means low security). This meant that when nursing students felt unsafe in clinical practice, higher self-efficacy could enable them to adopt better cognition and behavior when faced with nursing errors, which was in agreement with Ma et al.'s (27) results. However, our research context differs from that of Ma et al. We explored the relationships among several variables based on the clinical internship of nursing students, while Ma et al. focused on the innovative ability of nursing students. In clinical practice, a nursing error might cause real harm to patients, which could result in slower patient recovery, worsening of the condition, loss of function, and even threatening the patient's life (28). In general, nurses and nursing students are always the second victims of nursing errors (29, 30) because they might face blame and ridicule from nursing teachers, colleagues, classmates, and even themselves, which could result in a serious negative impact both on their physical and mental health, such as fatigue, fear, guilt, anxiety, and depression (5). These experiences can cause them to withdraw from future clinical practice due to the associated risks. Thus, when students felt unsafe in the real clinical environment, only those confident in completing the current task were willing to engage in practical operations and thus grow from possible errors. In contrast, the self-efficacy of students with high security had no significant influence on their error orientation because they did not need to worry about dealing with negative outcomes. These students were continuously engaged in practice and faced errors or near errors regardless of their confidence in performing tasks correctly.

The results showed that when the security of nursing students was low (high score), the positive effect of self-efficacy on the motivation for failure escaping was strengthened, which meant that when nursing students felt unsafe in clinical practice, they were more likely to avoid performing unfamiliar tasks to prevent failure, even if they were confident. As a result, they missed many opportunities to learn from new practices and errors. In summary, it is important to improve the sense of security among nursing students to enhance their participation in daily care, allowing them to learn more from real clinical experience and errors.

The results of this research also showed that the security of nursing students was crucial in their repeated attempts at nursing practice during internships, which allowed them to make continuous progress by facing problems, solving problems, and learning from errors instead of blindly avoiding risks. Thus, nursing educators should take measures to improve the security of nursing students. First, it is important to enhance knowledge and skills regarding nursing errors, including understanding what nursing errors are, how to prevent them on their own, and how to deal with them, which enables nursing students to minimize harm from nursing errors as much as possible and avoid panic when encountering them. Second, establishing a truly just and fair system for reporting adverse events is necessary to ensure that nursing students are not criticized, either directly or indirectly, after nursing errors occur. Third, timely psychological assessment and counseling should be provided to nursing students who experience nursing errors to mitigate the profound negative impact on their professional psychology. Finally, clinical teachers should receive training on the prevention, response, and handling of nursing errors, especially on how to treat nursing students correctly after errors occur so that nursing students can learn from their mistakes and not withdraw from future nursing practice.

This study had several strengths and limitations. One strength was the preliminary analysis of the impact mechanisms of self-efficacy, motivation, and security on error orientation in nursing students, a topic that has received little attention in previous research. However, the cross-sectional nature of this study limited the exploration of error orientation over different periods of the internship, which might vary due to the gradual accumulation of clinical nursing experience. Furthermore, this study can only indicate correlations, not causal relationships. Thus, longitudinal studies are necessary for the future exploration of the error orientation of nursing students to overcome these limitations.

## 9 Conclusion

Security moderated both the direct and indirect effects of self-efficacy and motivation for failure escaping on nursing students' error orientation. The findings suggest that interventions aimed at improving nursing students' motivation for success and security while reducing their motivation for failure-escaping may help enhance their attitudes and behaviors toward nursing errors in clinical practice.

## Data availability statement

The original contributions presented in the study are included in the article/supplementary material, further inquiries can be directed to the corresponding author.

## Ethics statement

The studies involving humans were approved by the Ethics Review Committee of Life Sciences in the First Affiliated Hospital of Zhengzhou University. The studies were conducted in accordance with the local legislation and institutional requirements. The Ethics Committee/Institutional Review Board waived the requirement of written informed consent for participation from the participants or the participants' legal guardians/next of kin because The survey questionnaire is distributed in electronic form and states that as long as the respondents complete this survey, they will be deemed to have agreed to participate in this study.

## Author contributions

YG: Conceptualization, Formal analysis, Funding acquisition, Methodology, Resources, Writing – original draft. WF: Investigation, Software, Writing – review & editing. XD: Data curation, Methodology, Writing – review & editing. CY: Investigation, Writing – review & editing. MW: Investigation, Writing – review & editing. HG: Investigation, Writing – review & editing. PL: Investigation, Writing – review & editing. KM: Conceptualization, Project administration, Supervision, Validation, Visualization, Writing – review & editing.
